# Editorial: The science and art of value in healthcare: Measures, voices and methods

**DOI:** 10.3389/fpubh.2022.1089091

**Published:** 2022-11-24

**Authors:** Jon A. Chilingerian, Stephen E. Chick, Gala True

**Affiliations:** ^1^Institute for Health Systems, Heller School for Social Policy and Management, Brandeis University, Waltham, MA, United States; ^2^Public Health and Community Medicine, Tufts University School of Medicine, Boston, MA, United States; ^3^INSEAD – Europe Campus, Fontainebleau, France; ^4^Department of Medicine, Section on Community and Population Medicine, Louisiana State University School of Medicine, New Orleans, LA, United States

**Keywords:** healthcare value, healthcare quality, healthcare performance, patient experience, caregiver wellness

Every country entrusts its population's health to autonomous medical practitioners and institutions. The health and wellness of the population depends on how well these clinical professionals and institutions perform (1–3). Understanding, evaluating, managing, and improving performance requires the measurement of value (4). So, how do we understand and define value?

Value is not about quality^1^ or technical outcomes alone, any more than value is about prices and costs alone—although both are constraining factors. Today “value” means the simultaneous pursuit of (1) improving patient outcomes, (2) improving patient experiences, (3) lowering long-term costs by reducing or eliminating waste, and (4) taking responsibility for the wellness of the caregiver workforce (5, 6). Given that definition, it becomes clear that when value improves, patients, caregivers, medical organizations, and payors benefit. Contrarily, when value deteriorates, so too do outcomes, patient experience, safety, efficiency, convenience, and patients' and health workers' wellness.

This Research Topic aims to better understand the science and art of high-value medical care for patients and populations, clinicians and staff, medical care delivery, and ecosystems. This includes measuring and assessing value in healthcare and how innovation, efficiency, and care redesign can improve value. An aligned understanding of value across healthcare stakeholders is fundamental to improving innovation, access, efficiency, payment and finance systems, and research vis-à-vis patient care.

We have organized the 12 publications in this Research Topic into five categories^2^:

1. Value creation for patients and populations (*n* = 5).
2. Value creation for the people working in medical care (*n* = 2).
3. Value creation for medical care organizations (*n* = 3).
4. Value creation for health systems (*n* = 1); and
5. Value creation for payors (*n* = 1).

The studies take place on three continents and in seven countries: viz., Australia, Canada, India, Oman, Spain, Switzerland, and the United States. These papers suggest that high-value healthcare may be emerging as an international trend. The five categories of value creation will be discussed in more detail in the following section.

## Value creation for patients and populations

Value for patients involves a patient's perception that the medical services offered benefited that patient in terms of outcomes and experience in relation to the sacrifices made to obtain those services. Durosini et al. report on a qualitative study protocol that uses focus groups and nominal group discussions to elicit lung cancer patients' views on the most important treatment options. Illustrating what scholars have stated, “value can only be defined by the ‘ultimate' customer” (7).

Hajjar and Kragen examine the use of telehealth during COVID-19 for a child with a chronic illness. Their work demonstrates the added value of telehealth in providing more timely communication and improved care coordination, ensuring person-centered care for families coping with chronic disease. Bhyat et al. report that after COVID-19, the lethargic utilization of telehealth in Canada changed from 4% utilization in 2019 to 14% in 2020, with almost 56% strongly satisfied. This points out the downside of looking for value too soon and the importance of comparative results to realizing patient value.

Goff et al. describe a protocol for a multi-method study intended to explore barriers and facilitators to value creation in a state-wide implementation of a population health program in Massachusetts for people with limited income and resources. Given the $1.8 billion USD investment, it makes sense that policymakers will benefit from protocols that support value creation.

Nanda et al. challenge health resource allocation based only on the global burden of disease weights and expert opinion. Studying two communities in India, they create community-derived disability weights for 14 illness conditions. Researchers found some significant differences between the two Indian states, but more importantly, a low correlation with the global burden of disease weights. Again, this case demonstrates the importance of patient input to understanding patient experience, especially when making resource allocation decisions.

## Value creation for medical practitioners, caregivers, and staff

Patient-centricity, as defined here, is a pivotal value designed to enhance patient outcomes and experience while motivating healthcare workers. This gives rise to two questions. As value-based healthcare is adopted and assimilated into medical care organizations, how has the drive for patient-centered care affected the wellness of healthcare professionals? And how do we mobilize and energize direct care staff to want to offer higher-value care?

Engen et al. report a systematic meta-review of value-based healthcare. They found two contrasting aspects of value-based care by differentiating job resources from job demands. Embedding people-centered values in the workplace and culture may be equally important as the drive for patient-centered values.

Another untapped source of value creation for caregivers is the power of social connections and relationships. Warfield et al. describe an action research project with employees and direct care staff at a residential home and the surrounding. Increasing organizational awareness of the relational strengths and weaknesses resulted in deeper engagement and resident-community involvement, thus effectuating both caregiver and care recipient wellbeing.

## Values creation for medical care organizations

How much value can healthcare managers add by adopting a high-value care strategy and challenging the status quo of core medical care processes? Bertke and Nufer suggest a three-step methodology for value creation with no trade-offs between quality and efficiency. Their approach reports significant improvements in patient satisfaction, readmission rates, shorter lengths of stay, and significantly lower costs.

Rodriguez et al. build execution into a high-value care strategy with ten lessons. The strategy measured quality and cost per patient for conditions ranging from breast and lung cancer to coronavirus. They estimated that the average time of a value-based management project could take between 18 and 24 months to implement at an average cost of @90.000 euros for a more complex medical condition care process improvement.

Majewski presents a case study of a non-traditional partnership between an Australian university and a primary healthcare service organization. Adopting a structured innovation tournament and a collaborative process resulted in sustainable value creation for relatively small investments.

## Values creation for a health system

Offering a behind-the-scenes analysis and review of Oman's health response to COVID-19, Khalili et al. highlight the challenges that all governments face when a “wicked problem”^3^ becomes part of everyone's lives. The courage to move forward by taking action with curfews, night store closures, and putting schools online, underscores the need to inspire and mobilize the community to create, not undermine, value.

## Values creation for third-party payors

In general, third-party payors also want to reduce or eliminate unnecessary services that do not improve health but increase per-unit costs/prices. The paper by Lorenz and Doonan explores outcomes and cost-savings resulting from patients with traumatic brain injury having access to multi-disciplinary rehabilitation after injury. Employing a societal model of value, the authors identified significant lifetime savings per patient, creating a compelling case for payors.

Success in commercial and non-commercial enterprises and their eco-systems requires providing higher value to end users for a fair price and at a reasonable cost to the organization (7, 9–11). In this editorial, we have assumed that high-value healthcare is an appropriate aim of medical organizations and health systems—time will tell if that assumption is valid. This Research Topic surfaces several important and challenging questions about value, needing more research and analytic case studies. Improvement should always be our goal, and we have only scratched the surface.

## Author contributions

JC and GT contributed to the design and analysis of results. SC made additional analysis. All authors contributed to the article and approved the submitted version.

## Conflict of interest

The authors declare that the research was conducted in the absence of any commercial or financial relationships that could be construed as a potential conflict of interest.

## Publisher's note

All claims expressed in this article are solely those of the authors and do not necessarily represent those of their affiliated organizations, or those of the publisher, the editors and the reviewers. Any product that may be evaluated in this article, or claim that may be made by its manufacturer, is not guaranteed or endorsed by the publisher.

## Footnotes

1 Quality of care has both objective dimensions, e.g., technical outcomes, as well as affective dimensions, such as patient satisfaction. Quality is best understood as a multidimensional construct, operationalized by the following variables: technical outcomes; overall patient experience and satisfaction; decision-making efficiency; relationships with caregivers and staff; and convenience and amenities (5).

2 There are other value creating stakeholders that are beyond the scope of this Research Topic. For example, there can be value creation for biotech and medical technology innovators, developing treatments and tools to benefit patients and clinicians. These suppliers should coordinate with care delivery to ensure compelling value is being created by reducing long-term costs and prices.

3 COVID-19 is an example of a wicked problem—a class of social system problems that are not well-formulated, the stakes are extremely high, there is ambiguity, there are no technical experts, and stakeholders have conflicting values (8).
